# Pediatric Implant of a Gold‐Coated Defibrillator due to Persistent Metal Hypersensitivity: Case Report

**DOI:** 10.1111/anec.70120

**Published:** 2025-10-21

**Authors:** Marcos Javier Duarte‐Sau, José Cruz Arzola‐Hernández, Anwar Hazael Gutiérrez‐García, Amalia Castro‐Rodríguez, Diana Rosales‐Mendoza

**Affiliations:** ^1^ Department of Electrophysiology Cardiology Hospital, 21st Century National Medical Center, Mexican Institute for Social Security Mexico City Mexico; ^2^ Department of Electrophysiology Cardiology Hospital #34, “Dr. Alfonso J. Treviño Treviño”, Mexican Institute of Social Security Monterrey Mexico; ^3^ Department of Cardiology Cardiology Hospital #34, “Dr. Alfonso J. Treviño Treviño”, Mexican Institute of Social Security Monterrey Mexico; ^4^ Department of Pathology Cardiology Hospital #34, “Dr. Alfonso J. Treviño Treviño”, Mexican Institute of Social Security Monterrey Mexico; ^5^ Department of Pediatric Cardiology Cardiology Hospital #34, “Dr. Alfonso J. Treviño Treviño”, Mexican Institute of Social Security Monterrey Mexico

**Keywords:** CIED infection, gold‐coated defibrillator, hypertrophic incisional scarring, ICD generator exposure, metal hypersensitivity

## Abstract

A 9‐year‐old boy presented with repeated exteriorization of four implantable cardioverter‐defibrillators (ICDs), despite changes in implantation site and the use of antimicrobial or polymeric envelopes. The initial device was placed following an episode of ventricular fibrillation, with imaging revealing non‐obstructive hypertrophic cardiomyopathy. Over subsequent procedures, he developed hypertrophic incisional scarring and granulomatous inflammation. Titanium hypersensitivity was confirmed via dermal testing, though all wound and blood cultures remained negative. After partial exteriorization of a fifth device, a gold‐coated ICD was implanted, with improved wound healing. This case underscores the need to consider allergic reactions to device materials when managing recurrent pocket complications.

## Graphical abstract

A 9‐year‐old with hypertrophic cardiomyopathy developed repeated defibrillator exteriorizations due to hypersensitivity, unresponsive to various devices and materials. Resolution was achieved with a custom gold‐coated device. This case underscores the diagnostic challenges and need for individualized, hypoallergenic strategies in managing rare cardiac device allergies, especially in pediatric patients.

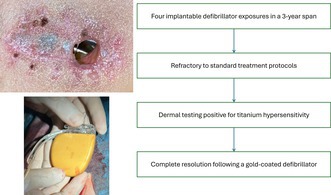

## 1 Introduction

Cardiac implantable electronic device (CIED) hypersensitivity reactions are extremely infrequent and often dismissed, as skin testing is frequently inconclusive and diagnosis is usually suspected only after excluding CIED infection (Slim et al. 2018). Various CIED components associated with allergic reactions have been described, including titanium (mainly used in generator casing), metal alloys (found in lead conductors), silicone, and polymers (used in leads), among others (Goli et al. 2012). We report a case of persistent CIED exteriorizations attributable to metal hypersensitivity in both subcutaneous and endovascular implantable cardiac defibrillators (ICD) at different implantation sites, which were non‐responsive to biocompatible polymers.

## 2 Case Report

**FIGURE 1:**
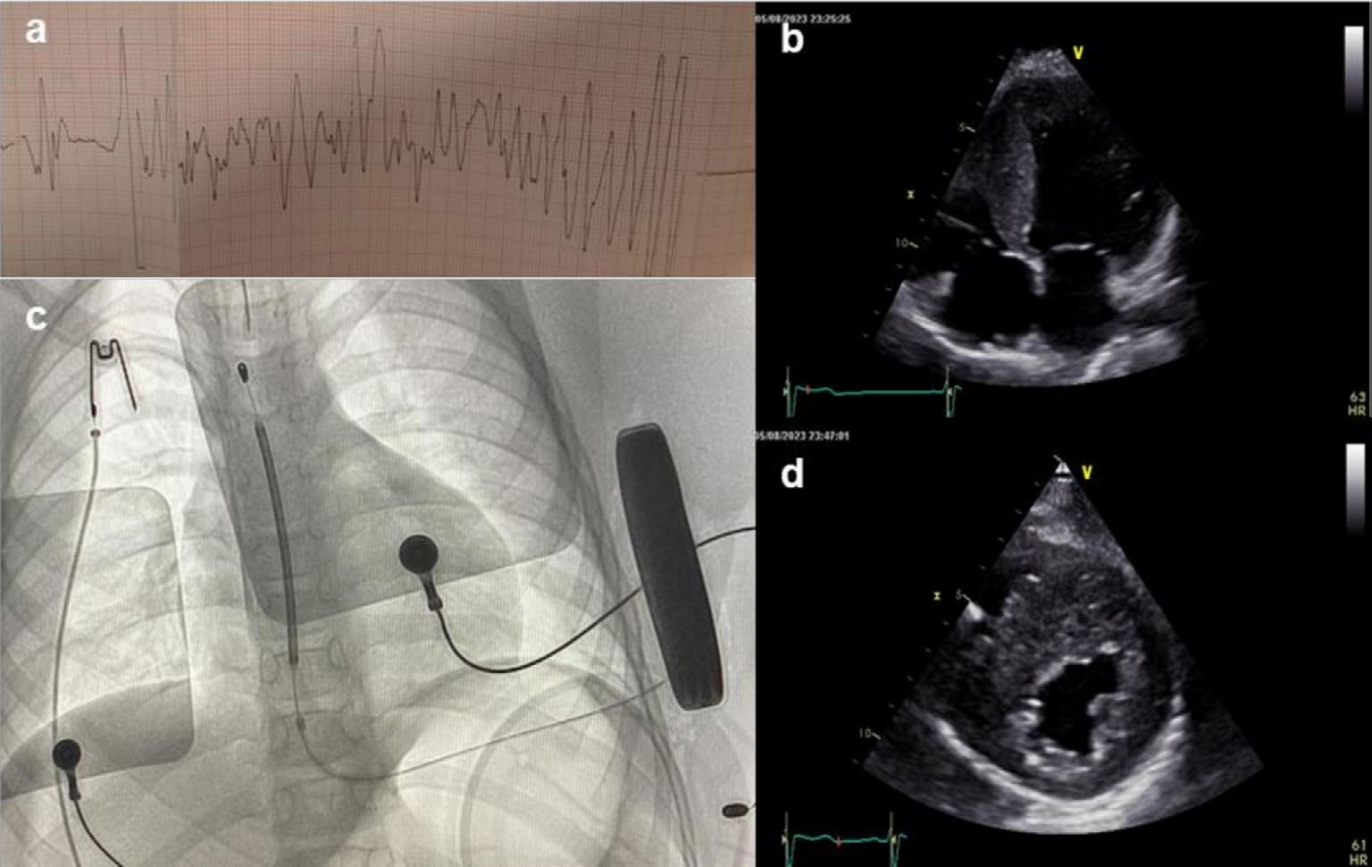
(a) Single‐lead electrocardiogram showing coarse ventricular fibrillation. (b) Apical four chamber view with asymmetrical septal hypertrophy. (c) Fluoroscopic anteroposterior thoracic view with a subcutaneous implantable cardioverter‐defibrillator. (d) Short axis view with the anterior septum majorly involved.

A 9‐year‐old male presented with recurrent exteriorization of four ICDs. His medical history included aborted cardiac death due to ventricular fibrillation at age 7 (Figure 1a). Baseline electrocardiogram, cardiac stress testing, and Holter monitoring appeared normal. Echocardiography revealed non‐obstructive hypertrophic cardiomyopathy (Figure 1b,d). A first subcutaneous ICD (S‐ICD) (Emblem MRI A219; Boston Scientific, Marlborough, MA) was implanted subcutaneously as secondary prevention (Figure 1c). Five months later, he developed hypertrophic incisional scarring (HIS) and swelling at the pocket site without purulent discharge, despite standard wound care. There was no systemic inflammatory response, as complete blood count and high‐sensitivity C‐reactive protein levels were within normal ranges, and the patient remained afebrile. Blood and wound cultures were negative.

**FIGURE 2:**
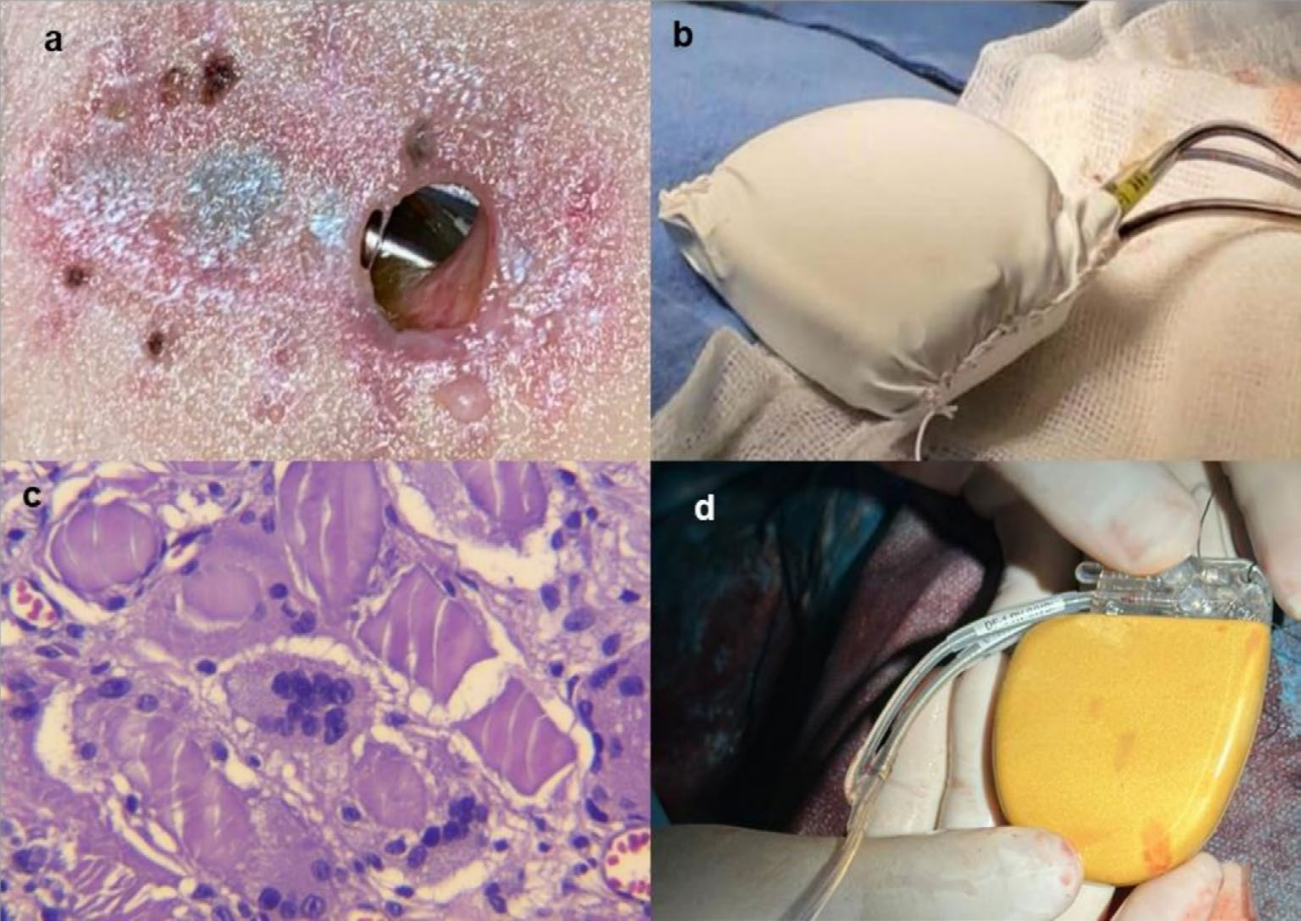
(a) Partial wound dehiscence and ICD generator exteriorization with hypertrophic incisional scarring. (b) Polytetrafluoroethylene‐wrapped ICD generator used in the fourth implant. (c) Chronic granulomatous inflammatory response with characteristic epithelioid cells with foreign material inside. (d) Gold‐coated ICD generator implanted.

He was then scheduled for complete generator removal and implantation of a new S‐ICD with a commercial antibacterial envelope (TYRX; Medtronic, Minneapolis, MN). One month later, the S‐ICD generator again exteriorized after developing HIS. A third endovascular ICD (Visia AF; Medtronic, Minneapolis, MN) was implanted via the left subclavian vein following removal of the second S‐ICD. He was readmitted 25 days post‐implantation due to wound dehiscence and HIS despite appropriate post‐procedure wound care, and generator exposure soon developed (Figure 2a). Blood and wound cultures were again negative, and complete removal of leads and generator was planned. A fourth contralateral ICD (same model with GORE‐TEX PRECLUDE) was implanted (Figure 2b). Upon suspicion of an ICD compound hypersensitivity reaction, the patient was referred for allergology and immunology consultation. Dermal patch tests for several ICD components and metals were performed, none of them reactive. Intravenous steroids and immunoglobulin were started, with partial wound healing observed. Pocket tissue biopsy revealed an inflammatory foreign body response (type IV hypersensitivity) with chronic granulomatous reaction and giant multinucleated cells (Figure 2c).

During 1 year of follow‐up, the fourth ICD became exposed. After explantation, a custom‐made 99% pure 18‐carat gold‐coated ICD (Cobalt XT DR, DDPA2D1G; Medtronic, Minneapolis, MN) was decided to be implanted via the left subclavian vein (Figure 2d). The last implant was reported as successful at a 2‐year follow‐up with no complications.

## 3 Discussion

An estimated incidence of CIED hypersensitivity reactions of 1 in 2000 implants has been described. Allergic reactions to ICD components are infrequent and diagnosed primarily after excluding CIED infection. Clinical presentation includes cutaneous signs common to both infection and hypersensitivity reactions, such as skin erosion and erythema. Diagnostic criteria have been proposed elsewhere (Robledo‐Nolasco et al. 2022).

Complete device removal and reimplantation with a non‐allergenic material coating has been suggested as the preferred treatment (Déry et al. 2002). Reasonable strategies to avoid this problem depend on the identification of the allergen. ICD, as in other CIEDs, consists of a pulse generator, electrode connectors, and leads. Depending on the manufacturer, several metal alloys and polymers are used. ICD generator encasing, pace‐sense electrodes, and shock coils are made from platinum‐iridium alloys sometimes coated with titanium nitrate. Lead connectors contain either stainless steel or a multi‐metal alloy including nickel, cobalt, chromium, and molybdenum. Lead insulators may be manufactured from various polymers such as ethylene tetrafluoroethylene, silicone, and polytetrafluoroethylene (PTFE) (Kealaher et al. 2023). In a 24‐patient European cohort, lymphocyte transformation tests assessing metal hypersensitivity to eight different metals showed positive results for titanium in 46% of patients (Manoušek et al. 2018). A non‐reactive or negative allergy patch test does not necessarily exclude titanium or other metal alloy hypersensitivity (Duarte et al. 2023).

To avoid contact with certain metal alloys, several wrapping materials may be employed. Custom‐made gold‐coated generators and PTFE membranes have been reported with favorable outcomes (Robledo‐Nolasco et al. 2022; Grosse Meininghaus et al. 2020). To the best of our knowledge, this is the first pediatric case of ICD implantation without clinical response to both subcutaneous and endovascular approaches, biocompatible polymer wrapping, and with the final resolution achieved through gold coating.

## 4 Conclusion

This case underscores the complexity and persistence of managing an uncommon presentation of CIED hypersensitivity. Recurrent device exteriorization occurred despite multiple implantations using different biocompatible materials, in the absence of infection. Awareness of this rare condition is essential, particularly in pediatric patients, where repeated surgical interventions pose heightened clinical and psychological burdens. In such scenarios, individualized solutions involving hypoallergenic materials may be required when conventional strategies prove ineffective.

## Author Contributions

The first draft of the manuscript was written by Marcos Javier Duarte‐Sau, and all authors commented on previous versions of the manuscript. Material preparation, data collection, and analysis were performed by Marcos Javier Duarte‐Sau and Hazael Gutiérrez‐García. Resources and interpretation of data were contributed by Amalia Castro‐Rodríguez and Diana Rosales‐Mendoza. Final supervision was revised by José Cruz Arzola‐Hernández. All authors read and approved the final manuscript.

## Consent

Written informed consent from the parents was provided prior to this case publication.

## Conflicts of Interest

The authors declare no conflicts of interest.

## Data Availability

The data that support the findings of this study are openly available in Research Square at https://doi.org/10.21203/rs.3.rs‐6926360/v1.
